# Disseminated *Neospora caninum* Encephalomyelitis and Myositis in a 3-Month-Old Cane Corso Puppy

**DOI:** 10.3390/vetsci11110544

**Published:** 2024-11-06

**Authors:** Abigail L. English, Joshuah B. Klutzke, Stephanie A. Thomovsky, Nobuko Wakamatsu

**Affiliations:** 1 Indiana Animal Disease Diagnostic Laboratory, West Lafayette, IN 47907, USA; 2 Department of Comparative Pathobiology, Purdue University, West Lafayette, IN 47907, USA; 3 Department of Veterinary Clinical Sciences, Purdue University, West Lafayette, IN 47907, USA

**Keywords:** canine, congenital, encephalitis, myositis, *Neospora*, paresis, paralysis, protozoal

## Abstract

We present a case of fatal disseminated neosporosis in a 3-month-old Cane Corso puppy. The puppy presented to the Purdue Veterinary Hospital emergency service with a 1-week history of progressive left hindlimb lameness. On presentation, the puppy was nonambulatory paraparetic. Signs progressed over two days to nonambulatory tetraparesis, and decreased to absent limb reflexes, cranial nerve deficits, and dull mentation. Blood work showed eosinophilia, elevated ALT, and extremely elevated creatine kinase at 36,000 IU/L (reference interval 22–491 IU/L). Two days after diagnostics were performed, the puppy experienced acute cardiac arrest, and the body was submitted for necropsy. At necropsy, skeletal muscle in all four limbs was diffusely pale tan. Tan streaks were disseminated through the diaphragm, abdominal wall, and myocardium. Histologically, Skeletal myocytes and cardiomyocytes were frequently degenerative, with abundant lymphohistiocytic inflammation and fibrosis. White matter within the brain and spinal cord was inflamed and contained frequent dilated myelin sheaths and spheroids. A few protozoal cysts were within the brain and skeletal myocytes. Antemortem antibody titers were positive for IgG against *Neospora caninum* (dilution titer of 1:4096, baseline 1:32), confirming protozoal cysts as *Neospora caninum*. Disseminated neosporosis is an uncommon but important clinical differential for ascending paresis in young dogs.

## Simple Summary

Disseminated neosporosis is an uncommon but important differential for progressive lameness in young dogs. *Neospora caninum* is a protozoan parasite with a two-host life cycle with canids as definitive hosts. Disease in immunocompetent adult dogs is rare; however, puppies may serve as aberrant intermediate hosts if infected congenitally. Affected puppies appear healthy at birth, and clinical disease usually develops from 3 to 9 weeks of age. Puppies show hindlimb weakness and paresis, which ascends and is generally fatal without treatment. A PCR of cerebrospinal fluid or affected tissues allows for antemortem diagnosis, though high IgG antibody titers are presumptively diagnostic in puppies with consistent clinical signs. A 3-month-old Cane Corso puppy presented to the Purdue Veterinary Hospital emergency service with a 1-week history of progressive left hindlimb lameness. The puppy was non-ambulatory paraparetic in both pelvic limbs at presentation. The puppy became tetraparetic, developed a dull mentation, cranial nerve deficits, and eventually suffered a cardiac arrest over the next four days. At necropsy, lymphohistiocytic inflammation was seen in the brain, spinal cord, myocardium, and skeletal muscle, with a few protozoal cysts seen in the brain and skeletal muscle. Antemortem titers returned positive for IgG (dilution titer of 1:4096, baseline 1:32), and *Neospora caninum* PCR of the skeletal muscle and brain was positive, with a Ct value of 22.09, confirming infection with *Neospora caninum*.

## 1. Introduction

*Neospora caninum* is a protozoal organism with a two-host life cycle. Dogs and wild canids are the definitive hosts, shedding oocysts in feces, while the primary intermediate hosts are cattle and other ruminants [1,2]. The most common and important manifestation of clinical disease is abortion in ruminants due to the vertical transmission of tachyzoites to the fetus in an infected dam [2]. The life cycle and pathogenesis of *N. caninum* is complex and not entirely understood. Canids are the only natural definitive hosts, shedding oocysts. These oocysts sporulate in the environment and are ingested by intermediate hosts. Oocysts then rupture and release sporozoites and bradyzoites, which convert to tachyzoites, replicate, and disseminate into tissues, including the gravid uterus. As host immunity develops, tachyzoites convert back to bradyzoites and encyst, mainly in neural tissue and skeletal muscle. These cysts may then be ingested by dogs eating raw or undercooked meat or aborted fetal materials, perpetuating the life cycle [1,2]. Definitive infection in adult dogs may also become chronic through tissue cyst development. Although dogs serve primarily as the definitive host for *N. caninum*, they may also serve as aberrant intermediate hosts, primarily in congenitally infected puppies [3]. Congenital infections may occur in puppies born to dams infected during pregnancy or to seropositive dams which undergo the re-activation of a chronic infection [4].

Clinical disease due to *N. caninum* infection is rare in immunocompetent adult dogs and cattle. While congenital infection in cattle usually results in abortion, congenital infection in dogs results in puppies which initially appear healthy, born with cysts and tachyzoites mainly within the brain, spinal cord, and muscle [1,3]. Clinical signs occur most commonly starting from 3 to 9 weeks of age, as the replication of tachyzoites continues and the immune response develops, worsening tissue destruction. Affected puppies show weakness, rigidity, and paralysis, which usually starts in and most severely affects the hindlimbs. If untreated, paralysis generally ascends, progressing to the forelimbs and neck followed by dysphagia and death [2,5]. Other manifestations vary by tissue affected and may include megaesophagus, myocarditis, respiratory disease, and skeletal muscle fibrosis leading to contracture or arthrogryposis [2].

Congenital infection in puppies is frequently fatal if untreated; prompt diagnosis is crucial. A definitive antemortem diagnosis is made through a PCR of CSF or affected tissues. Organisms may also be visualized and highlighted via immunohistochemistry but are rare in tissue, so the sensitivity of this method is low [2,5]. In a puppy with clinical signs of myositis or paralysis, presumptive diagnosis may be made if IgG antibody titers against *N. caninum* are positive, though clinically unaffected puppies may have positive titers [5]. The recognition of concerning clinical signs and rapid diagnosis and treatment are essential for survival and recovery in clinically affected puppies, as delayed treatment is associated with poor prognosis [5]. As such, it is important that clinicians can recognize the constellation of symptoms associated with congenital neosporosis in dogs. This case report presents a case of fatal disseminated neosporosis, causing ascending paresis and skeletal and cardiac myositis in a 3-month-old Cane Corso puppy.

## 2. Case Description

### 2.1. Clinical Findings

A 3-month-old male intact Cane Corso puppy weighing 12.25 kg presented to Purdue Veterinary Hospital emergency service with an approximately 1-week history of difficulty using the left hindlimb after having received a vaccination. On presentation to emergency, the patient was quiet, alert, and responsive. The patient was nonambulatory paraparetic with a proprioceptive ataxia in both pelvic limbs, the left being more affected than the right. Pelvic limb reflexes and perineal reflex were absent. The patient was neurolocalized to the L4-S3 spinal cord segments. Two days later, the patient presented to the neurology service for neurologic work-up. The patient had progressed and was now non-ambulatory tetraparetic. Reflexes were reduced in the thoracic limbs and absent in the pelvic limbs. Perineal reflex and anal tone were absent. The cutaneous trunci reflex was absent caudal to L1. The patient was mentally dull and had reduced to absent nasal sensation bilaterally and an absent gag reflex. The patient neurolocalized to multifocal central nervous system (CNS).

**Figure 1:**
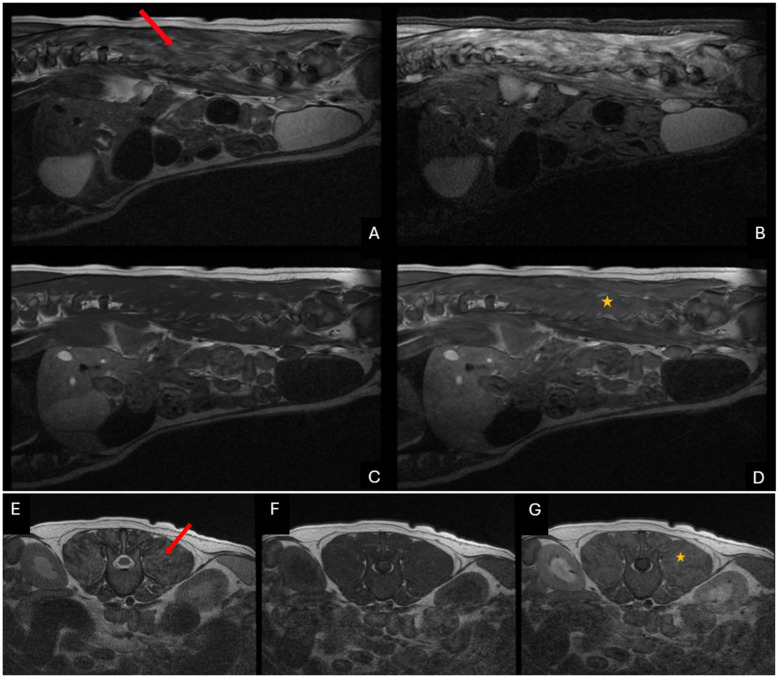
Parasagittal images of the lumbar spine (**A**–**D**) and transverse magnetic resonance images (**E**–**G**) at the level of the L4 vertebra. T2W images (**A**,**E**), T2W STIR images (**B**), T1W images (**C**,**F**) and T1W post-contrast images (**D**,**G**) are present. There are multifocal regions within the lumbar epaxial musculature that are hyperintense on T2W images (red arrow) and show heterogenous contrast enhancement (yellow star) on T1W post-contrast images.

Blood work, including complete blood count (CBC) and chemistry panel were performed, as was a brain and thoracic and lumbar spine magnetic resonance (MR) imaging and cerebrospinal fluid (CSF) analysis. The CBC showed an eosinophilia of 2.02 K/µL (reference interval 0.10–1.25 K/µL). On blood chemistry, ALT was elevated at 878 U/L (reference interval 3–69 U/L) and creatine kinase was elevated at 36,000 U/L (reference interval 22–491 U/L). The MRI showed multifocal, marked, diffuse T2-W hyperintensities and heterogenous contrast enhancement within epaxial musculature (Figure 1) and musculature of the head. The CSF analysis revealed an elevated protein of 212 mg/dL (reference interval < 25 mg/dL) and total nucleated cell count of 102 cells/µL (reference interval < 5 cells/µL) with 62% neutrophils.

Extensive additional testing was performed for infectious diseases, including serology enzyme-linked immunosorbent assay (ELISA) testing for *Dirofilaria immitis*, *Ehrlichia canis*, *E. ewengii*, *Borrelia burgdorferi*, *Anaplasma phagocytophilum*, and *A. platys*, enzyme immunoassay (EIA) urine antigen testing for *Blastomyces dermatitidis*, and serology immunofluorescent antibody tests (IFAT) for *Ehrlichia canis*, *Rickettsia rickettsii*, *Borrelia burgdorferi*, *Babesia canis*, *Cryptococcus neoformans*, *Toxoplasma gondii*, and *Neospora caninum*. Submitted antibody titers returned positive for IgG against *Neospora caninum*, with an IFAT dilution titer of 1:4096 (the baseline dilution is 1:32). All other antibody titers and antemortem infectious disease testing performed were negative. Due to the rapid progression of clinical signs, treatment was initiated before diagnostic results were received, and the patient was placed on broad spectrum antimicrobials including clindamycin (12.5 mg/kg twice daily) and trimethoprim/sulfamethoxazole (TMS, 20 mg/kg twice daily) as well as dexamethasone SP (0.14 mg/kg once daily) and gabapentin (8.3 mg/kg twice daily) to control inflammation and pain pending definitive diagnostic results. Two days after diagnostic testing was performed, the patient acutely experienced cardiac arrest. The body was submitted to the Purdue Animal Disease Diagnostic Laboratory for necropsy.

### 2.2. Gross and Histologic Findings

**Figure 2:**
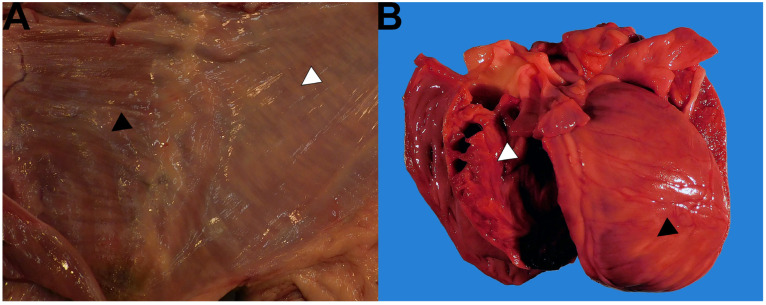
Gross photographs of the body wall, diaphragm, and heart. (**A**) Gross photograph of the body wall (white arrowhead) and diaphragm (black arrowhead). Skeletal muscle is firm and contains multifocal to coalescing pale tan to white streaks which extend throughout the muscle bodies on the cut section. (**B**) Gross photograph of the heart with opened left ventricle. Multiple pale tan streaks are visible on the epicardial surface (black arrowhead). The cut section of the ventricular wall (white arrowhead) is mottled tan.

At necropsy, skeletal muscle in all four limbs was diffusely pale tan and slightly firm. Well-defined pale tan to white streaks were disseminated within the muscles of the diaphragm, abdominal wall, and myocardium (Figure 2). The brain and spinal cord were grossly unremarkable. Along with standard tissues, multiple samples of axial and appendicular skeletal muscles, diaphragm, and spinal cord were fixed and examined histologically.

**Figure 3:**
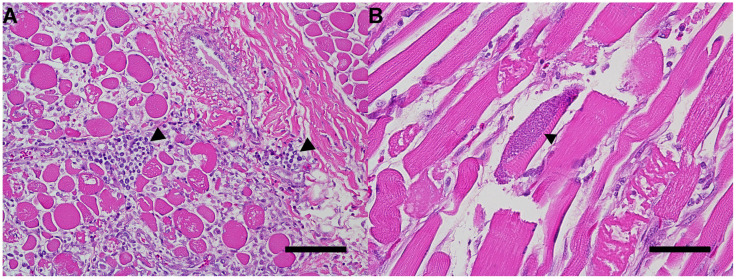
Photomicrographs of skeletal muscle. (**A**) Myocytes are separated by increased clear space (edema) and moderate inflammatory cells (arrowheads), primarily lymphocytes with fewer neutrophils. Sarcoplasm is frequently fragmented with loss of definition and necrosis of some fibers. H&E stain; bar = 100 µm. (**B**) A non-degenerate protozoal cyst containing numerous 1–2 µm diameter bradyzoites within the sarcoplasm of one myocyte (arrowhead). H&E stain; bar = 50 µm.

On histologic examination, cardiomyocytes and myocytes in multiple skeletal muscles were frequently degenerative and necrotic, with hypereosinophilic and fragmented sarcoplasm and increased dense collagen and fibroblasts. Abundant inflammatory cells, primarily lymphocytes, macrophages, and degenerate eosinophils and neutrophils, dissected between and replaced degenerating myocytes. Skeletal myocytes contained rare, approximately 22 µm diameter, well-defined, round basophilic structures containing numerous 1–2 µm, round, distinct basophilic bradyzoites (protozoal cysts, Figure 3).

**Figure 4:**
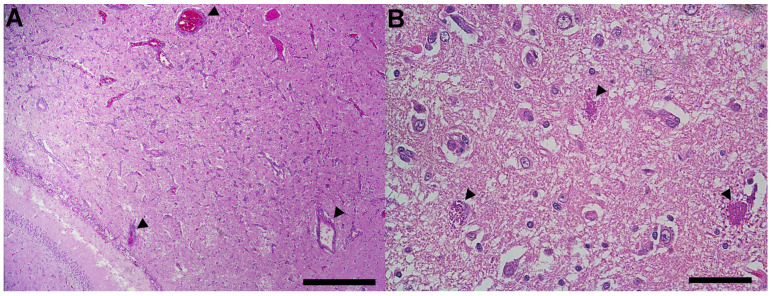
Photomicrographs of the cerebrum. (**A**) Low-power view of the focal white matter inflammation at the level of the hippocampus. Parenchyma is hypercellular with marked gliosis. Virchow–Robin spaces are expanded by moderate numbers of lymphocytes, plasma cells, and macrophages (arrowheads). H&E stain; bar = 500 µm. (**B**) Few degenerate protozoal cysts containing 1–2 µm diameter eosinophilic bradyzoites (arrowheads) are within the affected white matter. H&E stain; bar = 50 microns.

In the spinal cord, most significantly in the lumbar segment, gitter cells and few neutrophils infiltrated the white matter and more rarely the gray matter. Many myelin sheaths were dilated, commonly containing swollen and hypereosinophilic axons (spheroids) which were sometimes fragmented or absent, surrounded and replaced by gitter cells (digestion chambers). Similar changes were seen focally in the white matter at the level of the hippocampus, along with moderate expansion of the Virchow–Robin space by lymphocytes, plasma cells, and macrophages. A few degenerating protozoal cysts resembling those in the skeletal muscle were within areas of neuronal inflammation and necrosis (Figure 4). Real-time PCR performed on formalin-fixed, paraffin-embedded sections of the skeletal muscle and brain returned positive for *Neospora caninum* with a Ct value of 22.09.

## 3. Discussion

Necrotizing myositis, myocarditis, and encephalomyelitis seen histologically with intralesional protozoal cysts are consistent with disseminated protozoal infection, with protozoal cysts histologically consistent with *Neospora caninum* or *Toxoplasma gondii*. Positive IgG titers for *Neospora caninum* and a negative *Toxoplasma gondii* titer, along with positive *Neospora caninum* PCR, confirm the infection as systemic infection with *Neospora caninum*. While clinically unaffected puppies may have positive IgG titers for *Neospora caninum*, clinically affected puppies generally have high titers, as in this case; in one study of twelve dogs tested across three litters from the same dam, positive but unaffected animals had IgG titers ranging from 1:40 to 1:640, while their clinically affected sibling had a titer of 1:5120 [4].

Initial clinical signs of asymmetric paresis and lameness and progression to include cranial nerve deficits are unusual in congenital neospora infection. Both rapid progression of signs and delay of treatment are associated with poor prognosis in disseminated neosporosis [5]. Mildly to moderately elevated serum creatine kinase (CK) is common in dogs with neosporosis [5] and may raise clinical suspicion for the disease in dogs with neurologic signs while additional diagnostic tests are pending. However, a markedly elevated CK of 36,000 U/L, as seen in this case, is unusual; median CK elevation in positive dogs in one study was 1334 U/L, with the maximum recorded value at 3633 U/L [6]. This degree of elevation is most likely secondary to the diffuse, severe skeletal muscle involvement seen in this dog. The diffuse pallor of skeletal and cardiac muscle in this case correlates with histologic evidence of fibrosis and inflammation due to destruction of the skeletal and cardiomyocytes. As organisms are rarely seen within tissue, the severe myonecrosis is more likely considered to be secondary to inflammation rather than due to primary destruction by protozoal tachyzoites; however, organisms may have been initially more numerous but obscured or destroyed by chronic inflammation in this case.

Congenital neosporosis is an important clinical differential for hindlimb paresis in young puppies without a history of trauma or evidence of underlying skeletal lesions. Although the infection is congenital, the identification of a clinically affected puppy does not immediately indicate infection of all littermates. Puppies born to seropositive dams across multiple litters may sporadically develop clinical disease, though seropositive puppies do not always develop disease [4]. Dams with higher titers are more likely to transmit the organism to pups; dams in one study with immunofluorescent antibody titers of 1:200–1:800 had transmission rates of 20–44%, while those with positive titers less than 1:200 had transmission rates of 4–14% [4]. Given the severity and frequent mortality of clinical *N. caninum* infection, the serologic testing of littermates and additional monitoring or treatment of seropositive animals depending on antibody titers may be indicated. If identified early, treatment with clindamycin, sulfonamides, or ponazuril alone or in combination may be effective for decreasing or resolving clinical signs. Successfully treated animals should be assumed to be chronically infected, as bradyzoites within cysts are not susceptible to treatment [2,5]. Although appropriate treatment with clindamycin and TMS was initiated in this puppy before diagnostic testing results returned, the rapid progression of clinical signs precluded the determination of treatment efficacy.

## 4. Conclusions

The presentation of an ascending paresis with reflex deficits in a young puppy without evidence of trauma should raise suspicion of disseminated neosporosis, particularly if creatine kinase is elevated. Treatment should be initiated promptly in cases with a high index of suspicion for this disease, as delay can worsen prognosis for recovery. Congenital neospora transmission may not affect multiple puppies within one litter; however, given the morbidity of this disease, the testing of littermates and the monitoring or treatment of seropositive puppies is warranted if disseminated neosporosis is diagnosed in one member of the litter.

## Data Availability

This article includes all relevant study data.
